# Stem Cell Therapy in Injured Vocal Folds: A Three-Month Xenograft Analysis of Human Embryonic Stem Cells

**DOI:** 10.1155/2015/754876

**Published:** 2015-10-18

**Authors:** Bengt Svensson, Srinivasa R. Nagubothu, Christoffer Nord, Jessica Cedervall, Isabell Hultman, Lars Ährlund-Richter, Anna Tolf, Stellan Hertegård

**Affiliations:** ^1^Department of Public Health and Clinical Medicine, Unit of Clinical Research Center, Umeå University, 90187 Umeå, Sweden; ^2^Department of Otorhinolaryngology, 83183 Östersund, Sweden; ^3^Center for Hematology and Regenerative Medicine, Department of Laboratory Medicine, Karolinska Institutet, 14186 Stockholm, Sweden; ^4^Centre for Molecular Medicine, Umeå University, 90187 Umeå, Sweden; ^5^Department of Medical Biochemistry and Microbiology, Uppsala University, 75185 Uppsala, Sweden; ^6^Department of Women's and Children's Health, Karolinska Institutet, 17177 Stockholm, Sweden; ^7^Department of Immunology, Genetics, and Pathology, University Hospital, 75185 Uppsala, Sweden; ^8^Department of Clinical Sciences and Intervention, Karolinska Institutet, 14186 Stockholm, Sweden; ^9^Department of Otorhinolaryngology, Karolinska University Hospital, Huddinge, 14186 Stockholm, Sweden

## Abstract

We have previously shown that human embryonic stem cell (hESC) therapy to injured rabbit vocal folds (VFs) induces human tissue generation with regained VF vibratory capacity. The aims of this study were to test the sustainability of such effect and to what extent derivatives of the transplanted hESCs are propagated in the VFs. The VFs of 14 New Zealand rabbits were injured by a localized resection. HESCs were transplanted to 22 VFs which were analyzed for persistence of hESCs after six weeks and after three months. At three months, the VFs were also analyzed for viscoelasticity, measured as dynamic viscosity and elastic modulus, for the lamina propria (Lp) thickness and relative content of collagen type I. Three months after hESC cell therapy, the dynamic viscosity and elastic modulus of the hESC treated VFs were similar to normal controls and lower than untreated VFs (*p* ≤ 0.011). A normalized VF architecture, reduction in collagen type I, and Lp thickness were found compared with untreated VFs (*p* ≤ 0.031). At three months, no derivatives of hESCs were detected. HESCs transplanted to injured rabbit VFs restored the vibratory characteristics of the VFs, with maintained restored function for three months without remaining hESCs or derivatives.

## 1. Introduction

Tissue defects in a vocal fold (VF) heal with scar formation. The scar tissue causes stiffness in the lamina propria (Lp) which renders disturbed viscoelastic properties to the VF [1]. A scarred VF causes severe voice problems [2]. Treatment is difficult. Different injectable substances have been used to augment scarred VFs [3–12]. Several of these substances have rendered improved vibratory characteristics to the scarred VF, but presently there is no effective method to prevent VF scarring or to heal VF scars.

In xenograft studies, human mesenchymal stem cells (hMSCs) in different preparations have lately shown promising results in healing VF scars [13–18].

However, hMSCs have only been found to perform single divisions and have not been shown to have the capacity to differentiate into new tissue in the VFs [15, 16].

Since the first description of successful* in vitro* culture of human embryonic stem cells (hESCs), such cells have been recognized as to provide a potential resource for cell transplantations. HESCs are derived from the blastocyst of an early embryo and are unique in the sense that they have the capacity to self-renew but also that of pluripotency, that is, to differentiate into all cell types of the human body. In a previous study with human embryonic stem cells (hESCs) transplanted to injured VFs of rabbits, we found that hESCs can survive one month in a xenograft rabbit model and that they during this time can differentiate into fully developed epithelium, muscle, and cartilage tissue, adequately placed close to or interpositioned with the rabbit corresponding tissue, thus replacing lost rabbit VF tissue [19]. This study also showed that the VFs treated with hESCs gained a significantly improved viscoelastic function, measured as dynamic viscosity and elastic modulus, compared with untreated scarred VFs.

Using the same xenograft model, the aim of this study was to analyze in longer term, that is, three months, the sustainability of the improved healing in the hESC treated scarred rabbit VFs. Another aim was to examine the destiny of the hESCs and to explore if malignancies or teratomas would develop in the hESC transplanted VFs.

## 2. Material and Methods

The principal study design has been used by several investigators [7, 10, 13]. American and Swedish principles of laboratory animal care were followed. The experiment was approved by the Local Ethics Committee at the Karolinska Institutet and by the Regional Committee for Animal Experimentation, Stockholm, Sweden, S29-06, S115-08.

**Figure 1:**
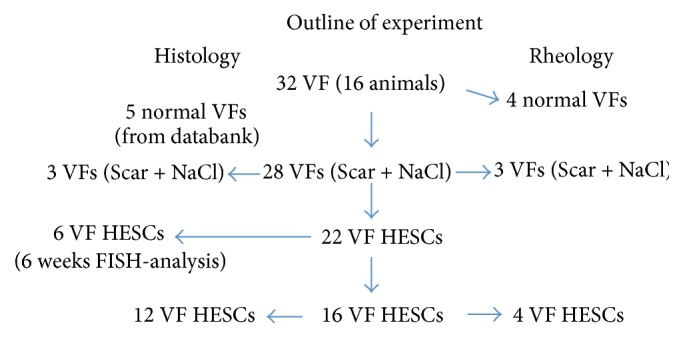


Sixteen female New Zealand white rabbits (bw 3.0 kg–4.0 kg) were used in the study. Twenty-eight VFs were operated on and the remaining four VFs were left as normal controls and used in the viscoelastic measurements. HESCs were transplanted to 22 of the 28 operated VFs and the remaining 6 VFs were left untreated, that is, scarred-untreated. Data for another five normal VFs were collected from a data bank from earlier experiments and were added in the histologic analyses, *n* = 32 + 5 [15, 16].  Figure 1 shows outline of experiment.

### 2.1. Vocal Fold Scarring

After premedication with glycopyrrolate (0.1 mg/kg s.c.) and Hypnorm (fentanyl citrate 0.3 mg/mL mixed with fluanizonum 10 mg/mL, 0.3 mL/kg i.m., Janssen Pharmaceutica, Beerse, Belgium) the animals were anaesthetized with diazepam (2 mg/kg i.v.). The laryngeal structures and the mobility of the cricoarytenoid joints were found normal at examination by means of a modified 4.0 mm paediatric laryngoscope (model 8576E, Karl Storz Endoskope, Tuttlingen, Germany) and a Storz-Hopkins 0° 2.7 mm rigid endoscope (model 7218A). The scarring procedure was performed with a 1.5 mm microcup forceps (MicroFrance Medtronic, Düsseldorf, Germany) excising the mucosa and the superficial layer of the thyroarytenoid muscle. A digital video recorded on a computer was made of the VFs before and after the operation (Richard Wolf video camera Number 5512 and a Canopus ADVC100 digital video converter, Reading, UK).

### 2.2. Human Embryonic Stem Cell Preparation and Characterization

The hESC line HS181 (46; XX) derived by the hESC network at Karolinska Institutet, Stockholm, Sweden, was kindly provided by Professor Hovatta et al. [20]. The HS 181 cells were maintained as previously described on mitotically inactivated, by 35 Gy irradiation, human foreskin fibroblasts [21]. HS181 cells corresponding to passage 33 were used in the study.

### 2.3. Vocal Fold hESC Transplantation

Three to four undifferentiated hESC colonies were dissected directly from the culture plates and aspirated by a 27-gauge needle of a laryngeal injector with a syringe of 1 mL saline (Medtronic Xomed, Inc., Jacksonville, FL). The 1 mL saline then contained approximately 10^4^ cells per 0.1 mL. Under video monitoring, using the 27-gauge needle Xomed laryngeal injector, the hESCs were transplanted by an injection of 0.1 mL of the solution into the lamina propria and/or the superficial part of the thyroarytenoid muscle of the scarred VF. The injection was carried out directly after the scar excision procedure. The correct injection site was stated by observed bulging of the VF corresponding to the injected volume.

To reduce rejection, the animals that received hESCs were treated with immunosuppressant Tacrolimus (TC), (0.05 mg/kg bw s.c.) every second day. The dose was based on the recommended dose/kg from the manufacturer and our previous experiments in rabbits [15, 16].

### 2.4. Sample Procurement

The animals were sacrificed with an overdose of pentobarbital sodium. Each larynx was dissected out and divided in the posterior midline. Three animals were sacrificed after 6 weeks and the VFs were analyzed for persistence of hESCs. The remaining thirteen animals were sacrificed after 3 months. Eleven hemilarynges were fresh frozen and kept at −70°C until viscoelastic analyses. The remaining hemilarynges were placed in 4% formaldehyde for histologic and antibody analyses.

### 2.5. Histologic Measurements

After fixation in 4% formaldehyde and 70% ethanol, the VFs removed from the larynges were further processed, dehydrated, and finally embedded in paraffin wax and cut into 5 *µ*m thick horizontal sections covering the whole thickness of each VF. Staining was made with hematoxylin-eosin (HE) for histologic analyses. Image analyses were made at 10x or 20x magnification after digitization of the microscopic images. The slides were blindly analyzed at the Department of Pathology, University Hospital, Uppsala, Sweden. Inter and intrareliability were assessed by blind reexamination of 10% of the slides, randomly chosen. The results were identical.

Twelve out of the 22 hESC treated VFs were prepared for histologic measurements at time point of three months. Comparisons with three scarred untreated (Scar + NaCl) VFs and with data for 5 normal VFs from the databank were performed [15, 16].

#### 2.5.1. Immunohistochemistry for Collagen Type I Staining

Staining was performed as previously described [13, 19]. Briefly, slides were deparaffinized in xylene, rehydrated in alcohol, and blocked in PBS containing 3% BSA. Slides were incubated with a primary antibody (antibody 6308, Abcam, Cambridge, UK), followed by incubation with a secondary antibody (nr.A21127 Jackson Immuno Research labs Inc., West Grove, PA). Sections were rehydrated in ethanol and xylene and mounted with Vectashield containing DAPI (Vector labs Inc., Burlingame, CA). The relative contents of collagen type I in the VFs were measured from the digitized stains after a color filtering and normalization process with Photoshop (version 8.0) and a custom made software that automatically summarizes color change for the collagen type I linked fluorescent antibody (Software by Hans Larsson, Karolinska Institutet, Department of Phoniatrics).

#### 2.5.2. Lamina Propria (Lp) Thickness

After being embedded in paraffin wax, each VF was cut in horizonal 5 *μ*m thick sections covering the whole VF. The right angle of the microtome toward the specimen was meticulously adjusted for each sample. The measurements of the Lp thickness were carried out on the digitized HE image representing the optimal level of each VF (custom made software by Hans Larsson, Karolinska Institutet). The Lp was measured at three spots representing each third of the VF. If a tendency of polyp formation was seen, the polyp was included in that section's measure point. Each single value was then used in the statistic evaluation.

The Lp of two of the hESC treated VFs were partly damaged in the cutting preparation processes and were left out in the Lp thickness measurements, resulting in *n* = 10 for the hESC treated VFs in the Lp thickness calculations.

#### 2.5.3. Hematoxylin-Eosin Staining for Analysis of General Fibrosis

The VFs were characterized into four categories depending on grade of scarring, that is, fibrosis. Grade a showed no or minimal signs of fibrosis. Grade b showed a focal or noncompact fibrosis in the Lp or superficial vocal muscle. Grade c showed a more compact fibrosis in the Lp and superficial muscle and Grade d showed a compact fibrosis in Lp and superficial muscle as well as fibrosis in the deeper part of the vocal muscle [16].

### 2.6. Fluorescence In Situ Hybridization (FISH-Analysis) for Persistence of Cell Derivatives from the Transplanted hESCs

Detection of human cells in the VFs was performed with a human DNA specific reference probe linked to a fluorescent molecule, that is, FISH-analysis.

The FISH-analysis was accomplished as previously described [19]. Briefly, slides were deparaffinized in xylene and rehydrated in alcohol, followed by pretreatment with pepsin and hybridization over night at 38°C with a human specific fluorescent probe (CEP X (DXZ1) Spectrum Green SRY Probe, human genomic DNA, Vysis Inc., Burlingame, CA). Six hESC treated VFs were analyzed after six weeks and 12 after three months.

### 2.7. Viscoelastic Measurements

The viscoelastic shear properties of VF tissue have been studied by several researchers [22, 23]. The parallel-plate rheometer in this experiment produces sinusoidal shear small amplitude oscillations at increasing frequency (within 0.01–15 Hz). We used an AR 2000 Rheometer (TA Instrument) with a stationary lower plate (8 mm diameter) separated by about 0.5 mm from a rotating upper plate. Tissue samples from the eleven fresh frozen VFs (4 scarred VFs injected with hESCs (HESC), 3 scarred injected with saline (Scar + NaCl), and 4 untreated, i.e., normal VFs (Normal)) were thawed in room temperature, dissected, and analyzed at 37°C in the parallel-plate rheometer. The samples included Lp and the superficial part of the thyroarytenoid muscle. The tissue was kept moist with saline during the measurements. All rheometric measurements were performed with a constant strain level transferred from the sample to the upper plate where it was measured with a linear variable displacement transducer. In this experiment, the response and reproducibility were stable up to 2-3 Hz. For higher frequencies, the results were not stable probably due to inertia of the measurement system as the tissue samples were not geometrically perfectly flat and did not completely fill out the 8 mm plate space. The dynamic viscosity (*η*′ in Pa·s) and elastic modulus (*G*′ in Pa) were derived as a function of frequency. Dynamic viscosity is a measure of material's resistance to shear flow. The elastic (storage) modulus (*G*′) represents a measure of material's stiffness in shear. As mentioned in this experiment, the gap between the plates was not completely filled with tissue. Thus the absolute levels of *η*′ and *G*′ may not be accurate. However, the same dissection procedure and amount of tissue were used for all samples which allows for comparison between the different groups.

**Figure 2:**
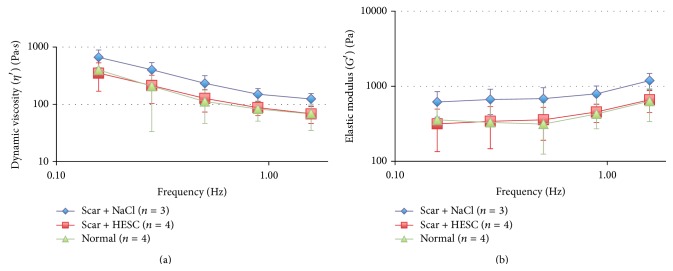
Rheological data showing (a) dynamic viscosity and (b) elastic modulus (as means ± 2 SD) versus frequency. Both were significantly reduced in the vocal folds (VFs) treated with human embryonic stem cells (hESCs) compared with untreated VFs (Scar + NaCl) (*p* = 0.011 and *p* < 0.001, resp.). Dynamic viscosity and elastic modulus of the hESC treated VFs did not significantly differ from normal VFs.

Measurements of *G*′ (Pa) and *η*′ (Pa·s) as functions of frequency, *f*, (in Hz) were plotted in log-log scale as shown in Figures 2(a) and 2(b). Curve-fitting regression was then performed for each curve to examine the relationships between *G*′ and *f* and between *η*′ and *f*. The obtained data were properly described using the quadric model rather than the linear one. The quadratic model was used for both *G*′ and *η*′, that is, log⁡⁡(*G*′  or  *η*′) = *B*_0_ + *B*_1_ · log⁡⁡(*f*), where *B*_0_, and *B*_1_ are coefficients of parameterization. The curve-fitting estimations, based on least-squares regression analysis, resulted in highly significant findings using the ANOVA *F* test in all cases (*G*′*p* = 0.001 and *η*′*p* = 0.006). The significant values of the *F* test suggested that the variation explained by the model was not due to chance. Goodness of fit was also estimated by the coefficient of determination, *R* ^2^. The *R* ^2^ statistic is a measure of the strength of association between the observed and model-predicted values for both log⁡⁡(*G*′) and log⁡⁡(*η*′). The values of *R* ^2^ were high for each regression model indicating goodness of fit (*R* ^2^ > 0.99 for both log⁡⁡(*G*′) and log⁡⁡(*η*′)).

### 2.8. Statistics

Differences between groups were assessed using Mann-Whitney *U* test for independent data. For the histologic measurements, each single value was included when differences between the various groups were estimated. Calculations, whether or not the dynamic viscosity and the elastic modulus, respectively, differed between normal, hESC treated VFs, and untreated scarred controls, were performed with the binomial test. In the regression analyses, the ANOVA *F* test was used. To calculate differences between groups in the analyses of general fibrosis, Fisher's exact test was used. Statistical significance was considered when *p* < 0.05.

## 3. Results

### 3.1. Viscoelastic Analyses

*Dynamic Viscosity, η*′* (Pa·s)*. Scarring significantly increased the dynamic viscosity, indicating stiffer folds, compared with the normal VFs (*p* = 0.006). Treatment with hESCs significantly decreased the dynamic viscosity compared with the untreated scarred controls (Scar + NaCl) (*p* = 0.011) and was not significantly different from the unscarred controls, that is, normal VFs (*p* = 0.4) (Figure 2(a)).

*Elastic Modulus, G*′* (Pa)*. Scarring also significantly increased the elastic modulus compared to normal VFs (*p* = 0.001). Treatment with hESCs significantly decreased the elastic modulus in comparison with the untreated scarred controls (Scar + NaCl) (*p* < 0.001). No significant difference was shown for the hESC treated VFs compared with the unscarred controls, that is, normal VFs (*p* = 0.4) (Figure 2(b)).

### 3.2. FISH-Analysis for Persistence of Transplanted hESC Derivatives

**Figure 3:**
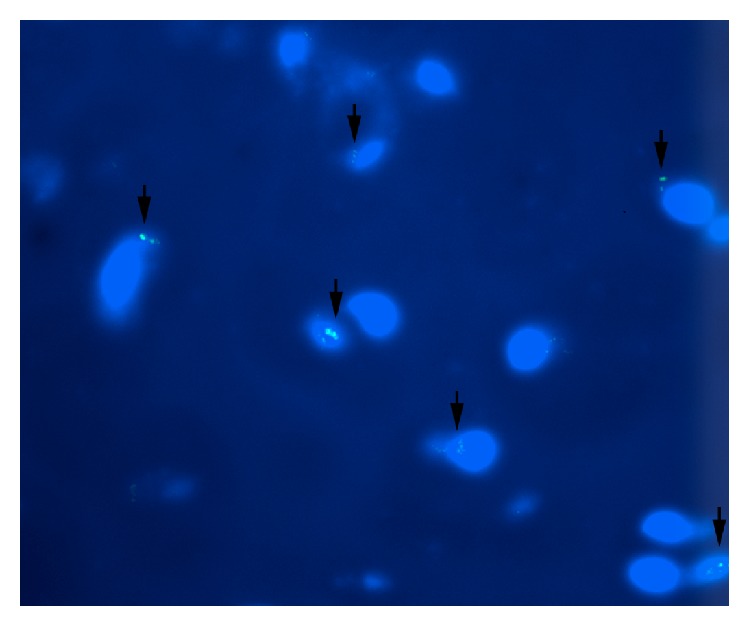
Fish staining (fluorescence in situ hybridization linked to a green fluorescent molecule; see text) showing hESCs in division at six weeks. Green enlightening represents human cells. Blue cells represent DAPI (4′,6-diamidino-2-phenylindole) fluorescent stain colored nuclei, in both rabbit and human cells. Black arrow marks human cells. 40x magnification.

Six weeks after the hESC injections, four out of six treated VFs showed presence of human cells as detected by FISH-analysis. Mitotic activity was rare and detected in 2 out of 6 VFs (Figure 3).

Three months after the hESC injections none of the 12 hESC treated VFs showed persistence of human cells or derivatives, as indicated by the lack of cells positive for the FISH analysis.

### 3.3. Histologic Analyses 

#### 3.3.1. Lamina Propria Thickness

**Figure 4:**
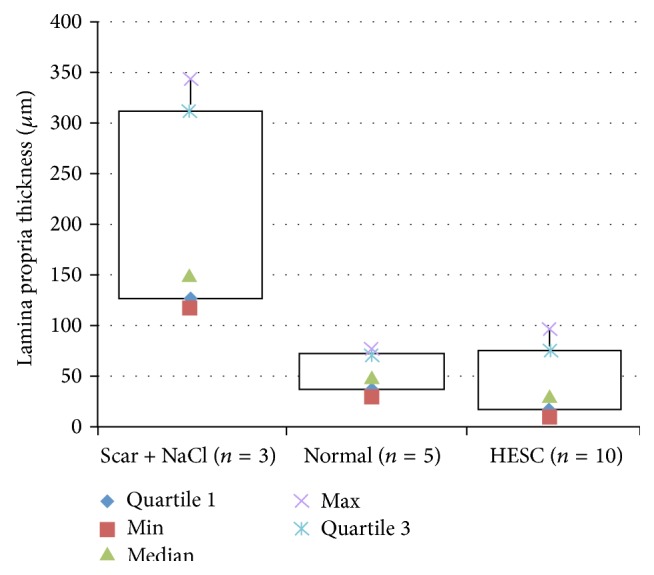
Lamina propria (Lp) thickness (*μ*m) is reduced in the vocal folds (VFs) treated with human embryonic stem cells (hESCs) compared with untreated scarred VFs (Scar + NaCl) (*p* < 0.001). The difference between hESC treated and normal VFs is nonsignificant.

The hESC treated VFs showed a significantly reduced Lp thickness compared with the scarred untreated VFs (*p* < 0.001). No significant difference was shown between hESC treated VFs and normal VFs (*p* > 0.05). The difference between untreated VFs and normal VFs was significant (*p* < 0.001) (Figure 4). The Lp thickness was performed blindly. Mean Inter and intrareliability were examined on 20% of the samples and the difference between the measurements was found to be less than 5% with a correlation coefficient of 0.99 at repeated measurements.

#### 3.3.2. Collagen Type I Staining

**Figure 5:**
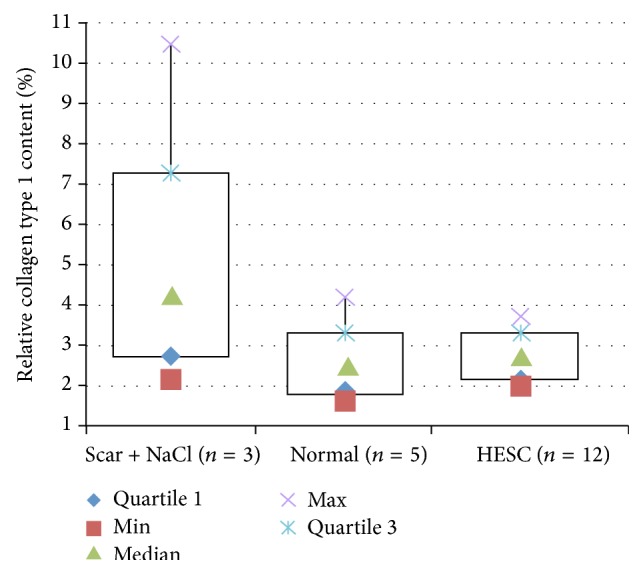
Collagen type I content (%). HESC treated vocal folds (VFs) show a reduction in collagen type I content compared with untreated scarred VFs (Scar + NaCl) (*p* = 0.031) and no significant difference to normal VFs.

The hESC treated VFs showed significantly reduced collagen type I in comparison with the scarred untreated VFs (*p* = 0.031). The difference between normal and hESC treated VFs was not significant (*p* > 0.05). The difference between normal and untreated VFs was significant (*p* = 0.037) (Figure 5).

#### 3.3.3. Hematoxylin-Eosin Staining for Analysis of General Fibrosis

**Figure 6:**
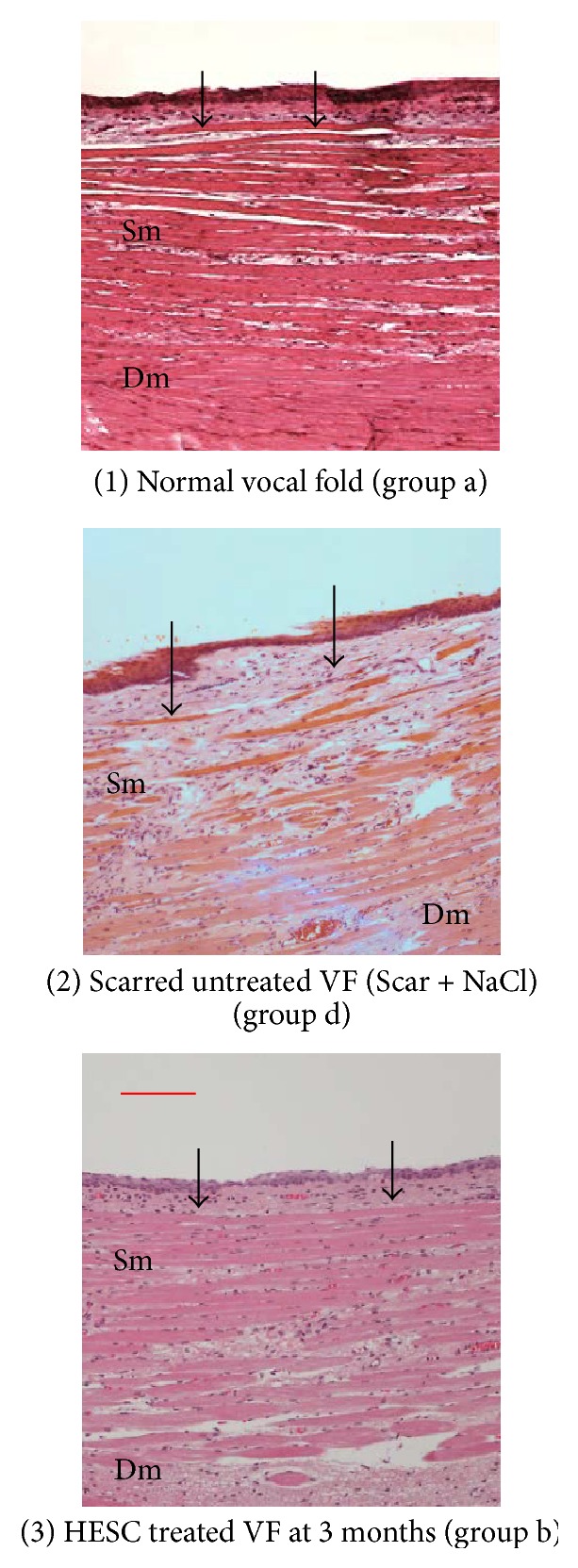
Longitudinal hematoxylin-eosin staining of the midmembranous part of (1) normal vocal fold (VF) with minimal loose connective tissue superficially under the lamina propria (Lp) with some inflammatory cells in the space of Lp, seen as black dots (group a, in the classification of general fibrosis; see Section 2.5.3). (2) Scarred untreated VF at three months showing compact connective tissue /fibrosis/ in deep Lp expanding into superficial muscle (Sm) and down into deep muscle (Dm). Plenteous inflammatory cells are spread in Sm and far into Dm (group d). (3) A hESC treated VF at three months showing minimal connective tissue in the Lp, slight loose connective tissue with limited inflammatory cells in Sm (group b). Arrows mark boarder between Lp and Sm. Scale bar is 100 *μ*m. The images slightly decolorized to visualize the greyish fibrosis.

Ten of the hESC treated VFs were placed in group b, one in each of groups a and c, and none in d (*n* = 12). Of the three scarred untreated VFs, two were placed in the c group and one in the d group (*n* = 3). When the a and b groups were compared with the c and d groups, the hESC treated VFs were placed in the a-b group and the untreated VFs in the c-d group (*p* < 0.011). Inter and intrareliability were identical in the blinded analyses (Figure 6).

#### 3.3.4. Hematoxylin-Eosin Staining for Malignancies or Teratomas

From the HE staining, there were no malignancies or teratomas found in the hESC transplanted VFs.

## 4. Discussion

The study is the second one published on hESC treatment of VFs. It shows that the hESC improved healing of scarred rabbit VFs seen after one month is sustainable over a longer period of time. Studies by our group and others have shown that VF scarring is established affecting both tissue viscoelasticity and histology within 3 months in a rabbit model. The specific maturation process of the scar may then take 3–6 months [15, 16, 24].

The derivatives of the transplanted hESCs were able to survive for six weeks but were not detected after three months in this rabbit model. Although a survival of hESC derivatives under the present level of detection cannot be ruled out, this indicates that the regenerated human tissue (epithelium, muscle, and cartilage) found after one month [19] can be expected to undergo apoptosis or rejection in a three-month period.

However, the present study shows that the vibratory characteristics, measured as dynamic viscosity and elastic modulus, were improved compared with untreated scarred VFs and showed statistically no difference to normal VFs after the three months. Moreover, in the histologic analyses no extensive scarring was found in the hESC treated VFs. In fact the collagen type I content and the Lp thickness values for the hESC treated VFs were not found different from the normal VFs. This indicates that lost regenerated human tissue is not replaced by scar tissue but by regeneration of compatible native rabbit tissue. By such factors, the apoptotic hESCs signal to the surrounding rabbit cells, in order to make them proliferate and not to create scar tissue, is unknown and is a challenge for further research.

Two hESC treated VFs did not show any human cells already after six weeks. These two VFs were classified to group b in the classification system of general fibrosis, that is, showing no more fibrosis than the average hESC treated VF. This suggests in this case an initiation of apoptosis already before six weeks, rather than a failed stem cell engraftment.

In all of the 12 VFs, the injected hESCs stayed in the injection site of the Lp and superficial vocal muscle. In none of the VFs, the hESCs were seen to migrate into the deeper part of the vocal muscle. This may be specific for VFs representing a relatively closed compartment [25].

None of the hESC treated VFs developed signs of malignancies or teratomas in this heterologous model. Notably, a human environment may behave differently. The general safety of hESCs however remains for future solutions.

In the study the immunosuppressant (Tacrolimus) was used to reduce the host versus graft reaction. The immunosuppressant was only administrated to the hESC treated VF individuals. This may have influenced the inflammatory reaction and also affected the hESCs. In a study with human mesenchymal stem cells comparing the healing process with and without immunosuppressant (Tacrolimus) we did not find any improved healing with immunosuppressant alone [26]. If the immunosuppressant has had any effect on the hESCs, it is reasonable to believe it has been negative. If so the hESCs have a potential to enhance their effect in an autologous environment.

Currently there is no ideal animal model representing the human VF. However, higher mammals as rabbits seem to have a rather similar VF healing capacity and pattern of scar formation as humans [24, 27, 28]. Therefore it seems reasonable to assume that the histologic and viscoelastic findings are transferable to the human VF.

## 5. Conclusion

We have previously shown that human embryonic stem cell (hESC) therapy to injured rabbit vocal folds (VFs) induces human tissue generation in the VFs with regained vibratory capacity of the VFs [19]. The present study shows that the hESC transplanted VFs maintained their restored function with improved vocal fold architecture as well as reduction in collagen and Lp thickness for three months although no residual hESCs or derivatives could be detected at this time point. At the three months, no malignancies or teratomas were revealed in the hESC treated VFs.
